# Risk perception in long-term evacuees of Futaba town, Fukushima: a cross-sectional study reveals greater concerns outside the prefecture, 12 years after the accident

**DOI:** 10.1093/jrr/rrae039

**Published:** 2024-06-11

**Authors:** Xu Xiao, Makiko Orita, Yuya Kashiwazaki, Hitomi Matsunaga, Thu Zar Win, Jacques Lochard, Noboru Takamura

**Affiliations:** Department of Global Health, Medicine and Welfare, Atomic Bomb Disease Institute, Nagasaki University, 1-12-4 Sakamoto, Nagasaki 852-8523, Japan; Department of Global Health, Medicine and Welfare, Atomic Bomb Disease Institute, Nagasaki University, 1-12-4 Sakamoto, Nagasaki 852-8523, Japan; Radiation Medical Science Center for the Fukushima Health Management Survey, Fukushima Medical University, 6th and 7th Floor, Medical Center for Fukushima “Life & Future Building (Mirai Bldg), Fukushima Medical University, 1 Hikarigaoka, Fukushima City, Fukushima 960-1295, Japan; Department of Global Health, Medicine and Welfare, Atomic Bomb Disease Institute, Nagasaki University, 1-12-4 Sakamoto, Nagasaki 852-8523, Japan; Department of Global Health, Medicine and Welfare, Atomic Bomb Disease Institute, Nagasaki University, 1-12-4 Sakamoto, Nagasaki 852-8523, Japan; Department of Global Health, Medicine and Welfare, Atomic Bomb Disease Institute, Nagasaki University Graduate School of Biomedical Sciences, 1-12-4 Sakamoto, Nagasaki 852-8523, Japan; Department of Global Health, Medicine and Welfare, Atomic Bomb Disease Institute, Nagasaki University, 1-12-4 Sakamoto, Nagasaki 852-8523, Japan; Department of Global Health, Medicine and Welfare, Atomic Bomb Disease Institute, Nagasaki University, 1-12-4 Sakamoto, Nagasaki 852-8523, Japan

**Keywords:** Fukushima Daiichi Nuclear Power Plant accident, long-term evacuation, radiation risk perception, risk communication, evacuation destination, post-disaster recovery

## Abstract

For over 12 years since the 2011 East Japan Earthquake, the decontamination of radioactive materials is still incomplete. Although evacuation orders had been lifted in ~15% of Futaba town, the site of the Fukushima Daiichi Nuclear Power Plant, by August 2022, anxiety regarding the effects of nuclear radiation persists among evacuees, and their intention to return (ITR) remains low. As of August 2023, only 90 residents lived there. As the only town with government functions relocated outside Fukushima Prefecture, Futaba has more residents who evacuated outside the prefecture. Although numerous factors affect risk perception and ITR to the place of previous residence, the impact of evacuation destination on risk perception remains unknown. Therefore, this study aimed to evaluate the impact of evacuation destination on radiation risk perception. In 2022, a survey was conducted on 404 evacuees aged >18 years. The responses were compared between groups outside and inside Fukushima using the chi-square test and multivariate logistic regression analysis. Significant relationships were found between the evacuation destination and risk perception of genetic effects in the next generation (odds ratio [OR] = 1.92, 95% confidence interval [CI]: 1.15–3.20) and of the health effects of radiation (OR = 1.76, 95% CI: 1.10–2.84), which were both higher in those who had evacuated outside Fukushima. These findings stress the importance of evacuation destination choice and information access for evacuees’ risk perception. Enhanced education and support efforts are necessary to help evacuees not only in Fukushima but also throughout Japan.

## INTRODUCTION

The explosion at the Tokyo Electric Power Company’s Fukushima Daiichi Nuclear Power Plant (FDNPP), which was the result of the Great East Japan Earthquake occurring on 11 March 2011, released various radioactive materials into the atmosphere and prompted the evacuation of residents in 12 affected municipalities in and around Fukushima Prefecture. The entire area of Futaba town, the site of the FDNPP, was under a mandatory evacuation order following the nuclear accident. Compared with other affected towns, which were all taken over by municipalities inside Fukushima Prefecture, the evacuation of Futaba town was more dispersed. The mayor arranged evacuation to Saitama Prefecture, which is 210 km from Futaba town, and also transferred government functions to Saitama Prefecture. Futaba is the only town for which the government was transferred outside of Fukushima Prefecture [1]. A substantial number of residents evacuated to a location outside Fukushima Prefecture, constituting 41% of the total population as of 31 August 2023, which is significantly higher than that for other towns [2]. The widespread location of evacuees has complicated communication with the government of Futaba town, especially in regard to radiation matters.

As of August 2022, nearly 12 years after the disaster (and in conjunction with the northeastern districts to which people were permitted to return in 2020), evacuation orders were lifted in ~15% of Futaba town (7.8 km^2^). Residents started to return, but as of 1 August 2023, only 90 people (1.6% of 5484 registered residents at the end of July 2023) were living there, including evacuees and newcomers [3], highlighting challenges in the recovery process. Previous studies, such as that by Matsunaga *et al.* [4] found correlations between anxiety levels and low return rates in evacuees in 2021 [4].

Futaba town restored its municipal functions in September 2022 and actively pursued urban development with the aim of increasing its population to 2000 by 2030 [5]. One crucial aspect of this recovery is the dissemination of accurate radiation information, fostering what the International Commission on Radiological Protection defines as a ‘practical radiation culture’, which is defined as ‘the provision of resources aimed at improving the knowledge and skills enabling citizens to make well-informed choices and behave wisely in situations involving potential or actual exposures to ionizing radiation.’ [6]. This involves providing resources to empower residents to make informed decisions about potential radiation exposure.

Previous surveys in other towns have shown that, compared with those living inside, residents living outside town limits express higher levels of health anxiety regarding radiation exposure, as well as poorer mental health. They are also less informed about official channels for information on nuclear radiation [7]. Risk perception may be related to the region where the residents are located. Given this background, we conducted a questionnaire survey in June 2022 to obtain insights into how information should be disseminated to evacuees living outside the prefecture.

## MATERIALS AND METHODS

The survey comprised self-reported questionnaire items on demographics, radiation risk perception, interest in radiation information, intention to return (ITR), and concerns about the revival of Futaba town. We also investigated the current status of residents’ regular access to hospitals. Because there was only one clinic in Futaba town until 2024 [8]; therefore, regular hospital visits may also impact evacuees’ intention to return. Strengthening the support for this group is crucial. Mental health was evaluated using the Mental Component Summary (MCS) of the Health-related Quality of Life Short Form-8. To prevent the possibility of duplicate responses, we included instructions in the questionnaire stating that each questionnaire should be completed by the same individual, ensuring one questionnaire per resident.

As of the latest data before the survey, on May 31, 2022, there were 5582 residents holding Futaba town household registration, with 2684 men and 2898 women, totaling 2179 households [9]. With the assistance of the town office, we attached questionnaires to public relations magazines sent by the Futaba government to each household in June 2022. Each household received two questionnaires. In total, 4358 questionnaires were distributed.

We received only 496 responses; of them, excluding 15 responses that did not indicate place of residence, 156 responses were from evacuees outside the prefecture, and 325 responses were from inside the prefecture, with a ratio of 0.48:1. According to the latest population data from the government of Futaba town prior to this survey, as of May 31, 2022, there were 2726 evacuees residing outside Fukushima Prefecture and 3954 evacuees inside the prefecture, with a ratio of 0.69:1 [10]. The response of evacuees outside the prefecture was lower. After excluding missing and contradictory information, we collected 404 valid responses from individuals aged >18 years who had active household registration in Futaba town. Of the participants, 130 and 274 had evacuated to a location outside and inside Fukushima Prefecture, respectively.

Data were analyzed with IBM SPSS Statistics (version 25; SPSS Japan, Tokyo, Japan) using the chi-square test and logistic regression analysis, and *P*-values <0.05 were considered statistically significant.

## RESULTS AND DISCUSSION

Table 1 shows the participants’ sociodemographic characteristics and the results of the chi-square test for all evacuees. Sex, age, employment, and living with children did not differ between inside and outside Fukushima, but there were more regular hospital visits for evacuees inside Fukushima. ITR was remarkably low whether residing inside or outside Fukushima (10.4%, *n* = 42), and no difference was found between outside and inside the prefecture. Regarding MCS scores, 58.4% (*n* = 236) of the evacuees scored below the Japanese average (50 ± 10), with no significant difference between them, indicating worse mental health. Regarding risk perception, more than half of all evacuees expressed anxiety about potential health (56.2%, *n* = 227) and genetic risks (60.0%, *n* = 242) from radiation exposure. Comparing evacuees to outside and inside the prefecture, a statistically significant difference was found, with a higher percentage of evacuees to outside the prefecture expressing anxiety about the health effects of radiation exposure (66.2% vs 51.5%, respectively; *P* = 0.005) and genetic effects in the next generation (71.5% vs 54.4%, *P* = 0.001). In addition, 69.6% (*n* = 281) of the evacuees indicated concern about the safety of tap water in Futaba town, and 65.1% (*n* = 263) about the scheduled discharge of treated water, which is filtrated by Advanced Liquid Processing System in August 2023; moreover, 69.6% (*n* = 281) expressed an interest in obtaining more information about treated water. No significant differences were found between inside and outside the prefecture in this particular aspect. In addition, evacuees to outside Fukushima were less aware of the radiation consultation center in Futaba town than were those inside the prefecture (65.4% vs 54.7%, respectively; *P* = 0.043). Nevertheless, the majority of evacuees (73.5%, *n* = 297) indicated anticipation about the revival of Futaba town. Approximately half of all evacuees expected workplace redevelopment (57.2%, *n* = 231), farmland redevelopment (49.8%, *n* = 201), and residential area rebuilding (55.7%, *n* = 225). No statistically significant differences were found between evacuees living inside and outside the prefecture.

**Table 1:** Sociodemographic characteristics, risk perception, and expectations for town revival of study participants according to evacuation destination

**Demographic variable**		**Evacuation destination**		***P* value**
		**Outside Fukushima (*n* = 130)**	**Inside Fukushima (*n* = 274)**	
Sex	Male	68 (52.3%)	143 (52.2%)	0.982
	Female	62 (47.7%)	131 (47.8%)	
Age (years)	< 60	31 (23.8%)	56 (20.4%)	0.436
	≥ 60	99 (79.2%)	218 (79.6%)	
Employed	Yes	31 (23.8%)	71 (25.9%)	0.665
	No	99 (76.2%)	203 (74.1%)	
Regular hospital visits	Yes	99 (76.2%)	231 (84.3%)	0.048^*^
	No	31 (23.8%)	43 (15.7%)	
Living with children aged <18 y	Yes	23 (17.7%)	35 (12.8%)	0.188
	No	107 (82.3%)	239 (87.2%)	
Intention to return	Intended	12 (9.2%)	30 (10.9%)	0.738
	Unsure	38 (29.2%)	86 (31.4%)	
	not intended	80 (61.5%)	158 (57.7%)	
Mental Component Summary	< 50	78 (60.0%)	158 (57.7%)	0.656
	≥ 50	52 (40.0%)	116 (42.3%)	
**Risk perception variable**				
Aware of radiation consultation center in Futaba town	Yes No	45 (34.6%) 85 (65.4%)	124 (45.3%) 150 (54.7%)	0.043^*^
Wish to acquire knowledge about nuclear radiation	Yes No	78 (60.0%) 52 (40.0%)	180 (65.7%) 94 (34.3%)	0.266
Anxious about discharge of treated water	Yes No	93 (71.5%) 37 (28.5%)	170 (62.0%) 104 (38%)	0.061
Wish to acquire knowledge about treated water	Yes No	96 (73.8%) 34 (26.2%)	185 (67.5%) 89 (32.5%)	0.197
Anxious about drinking tap water in Futaba town	Yes No	95 (73.1%) 35 (26.9%)	186 (67.9%) 88 (32.1%)	0.289
Anxious about the health effects of radiation exposure	Yes No	86 (66.2%) 130 (33.8%)	141 (51.5%) 274 (48.5%)	0.005^*^
Anxious about the genetic effects in the next generation	Yes No	93 (71.5%) 37 (28.5%)	149 (54.4%) 125 (45.6%)	0.001^*^
**Expectations for revival**				
Expect redevelopment of workplaces	Yes No	73 (56.2%) 57 (43.8%)	158 (57.7%) 116 (42.3%)	0.774
Expect redevelopment of farmland	Yes No	64 (49.2%) 66 (50.8%)	137 (50.0%) 137 (50.0%)	0.266
Expect rebuilding in residential areas	Yes No	69 (53.1%) 61 (46.9%)	156 (56.9%) 118 (43.1%)	0.466
Expect revival	Yes No	90 (69.2%) 40 (30.8%)	207 (75.5%) 67 (24.5%)	0.179

Note: chi-square test. ^*^: indicates a significant difference.

Table 2 shows the multivariate logistic regression analysis for anxiety about the genetic effects of radiation exposure and anxiety about the health effects of radiation exposure. The selection of explanatory variables was examined through chi-square tests, as detailed in Tables S1 and S2. The variance inflation factor (VIF) of all selected variances was much < 10 (all VIF values were between 1 and 2), and there was no serious multicollinearity.

**Table 2:** Multivariate logistic regression analyses for anxiety about the genetic effects of radiation exposure and anxiety about the health effects of radiation exposure

**Variable**	**Reference**	**Anxiety about genetic effects**	**Anxiety about health effects**
		**OR (95% CI)**	
Evacuation location	Outside/Inside Fukushima	1.92^*^ (1.15–3.20)	1.76^*^ (1.10–2.84)
Living with children <18 y	Y/N	0.53 (0.26–1.07)	/
Mental Component Summary	<50/≥50	2.65^*^^*^ (1.67–4.22)	2.63^*^ (1.69–4.09)
Aware of radiation consultation center in Futaba Town	N/Y	3.59^*^^*^ (2.25–5.74)	2.10^*^^*^ (1.34–3.26)
Wish to acquire knowledge about nuclear radiation	Y/N	1.12 (0.62–2.00)	1.64 (0.96–2.83)
Wish to acquire knowledge about treated water	Y/N	3.72^*^^*^ (2.02–6.84)	2.82^*^^*^ (1.61–4.95)
Expect town revival	Y/N	0.39^*^^*^ (0.22–0.68)	0.61 (0.37–1.01)

Note: logistic regression analysis. Y: yes; N: no; OR: odds ratio; CI: confidence interval; ^*^: *P* < 0.05; ^*^^*^: *P* < 0.001.

A major finding of this study was the presence of a significant relationship between evacuation destination and risk perception of the health effects of radiation exposure (odds ratio [OR] = 1.76, 95% confidence interval [CI]: 1.10–2.84) and of the genetic effects in the next generation (OR = 1.92, 95% CI: 1.15–3.20) after adjusting for living with children under 18 years old, which were both higher among those who had evacuated outside Fukushima (Table 2). Evacuee seekers with poorer mental health (MCS < 50) also showed greater concerns about genetic risk effects (OR = 2.65, 95% CI: 1.67–4.22) and health risk effects (OR = 2.63, 95% CI: 1.69–4.09). Evacuees unaware of the existence of a consultation center in Futaba town (OR = 3.59, 95% CI: 2.25–5.74) and who wished to acquire knowledge about treated water (OR = 2.10, 95% CI: 1.34–3.26) were generally concerned about genetic and health effects. There was a negative correlation between the expected revival of the town and genetic effects in the next generation (OR = 0.39, 95% CI: 0.22–0.67).

The United Nations Scientific Committee on the Effects of Atomic Radiation 2020 report states that there is no credible evidence of any health or genetic effects of radiation exposure (e.g. cancer, congenital abnormalities, stillbirth, premature birth, low birth weight) on the general population following the FDNPP accident [11]. In the previous study of other towns and villages, where evacuation orders were lifted much earlier, we also found that almost half of the residents were still concerned about genetic risks, even though more than a decade had passed since the accident [12, 13]. These findings suggest that heightened risk perceptions associated with nuclear disasters may persist over an extended period among residents of Futaba town.

This study highlights a significant correlation between evacuation destination and heightened anxiety regarding genetic and health effects of radiation exposure, which was higher among those who evacuated outside Fukushima as compared with those who evacuated inside Fukushima. During a nuclear disaster, an individual’s choices in obtaining information and trusting information sources have a direct influence on their risk perception. A previous study reported that participating in radiation health seminars can help alleviate anxiety about radiation risks [14]. The Futaba town government has been cooperating with nuclear radiation experts from various universities to conduct periodic nuclear radiation lectures for evacuees, explain the current situation of the town, provide personal dosimeter rental services, and offer internal radiation dose monitoring. Since the present findings indicated evacuees outside Fukushima were less aware of the radiation consultation center in Futaba town, and those unaware of the consultation center in Futaba town commonly expressed concerns about genetic and health impacts, we believed that extending these educational activities to those who evacuated outside Fukushima is necessary.

The main limitation of this study was the very few responses. This is similar to the historically low response rates observed in previous studies of Fukushima evacuees, mostly consisting of elderly individuals [15]. The aftermath of the 2011 disaster resulted in stakeholders, including government officials, academic institutions, and town councils, conducting surveys targeting residents. Over time, survey fatigue naturally occurred, especially since voluntary questionnaires, compared with mandatory government-led surveys, typically yield a response rate of 20% [16]. The spontaneous nature of responses introduces selection bias. Although we could not compare the attributes of evacuees for whom responses were not obtained, such verification was conducted using the Prefecture Health Survey [17]. Response rates from employed individuals and those with poor mental health tend to be lower, and residents interested in returning to or are attached to their hometown are more likely to participate. To capture a wider range of evacuee demographics, future research should reduce the burden on the respondents as much as possible. Online responses are also considered. Over 85% of the town’s areas are categorized as difficult-to-return zones, and these environmental conditions likely influence the intention to return. Finally, this was a cross-sectional study, and we could not establish causal relationships. Whereas most evacuees outside Fukushima prefecture were government-organized, we cannot assert that the evacuation location leads to different risk perceptions, since there is also the possibility of choosing different evacuation locations due to varying risk perceptions.

Nevertheless, our research still stresses the importance of information access for evacuees’ risk perception. It is vital that accurate information about radiation be disseminated to evacuees while facilitating reconstruction of the town. This approach can address evacuees’ risk perceptions about radiation while simultaneously supporting the rebuilding of the community. These findings suggest that there are enduring challenges for post-disaster recovery, and that radiation education is needed not only in Fukushima Prefecture, but also throughout Japan.

## Supplementary Material

TableS1-clean_rrae039

TableS2-clean_rrae039

## PRESENTATION AT A CONFERENCE

To be delivered as an oral presentation at the 94th Annual Meeting of the Japanese Society for Hygiene (7–9 March 2024).

## References

[1] Secretary and Public Relations Section, Futaba Town Office, Mayor’s Activities. 73-4 Chonishi, Nagatsuka, Futaba Town, Futaba District, Fukushima Prefecture. https://www.town.fukushima-futaba.lg.jp/item/5449.htm (29 January 2024, date last accessed). (in Japanese).

[2] Family Registration and Taxation Division, Futaba Town Office. Evacuation Status (as of August 31, 2023). 73-4 Chonishi, Nagatsuka, Futaba Town, Futaba District, Fukushima Prefecture. https://www.town.fukushima-futaba.lg.jp/10592.htm (29 January 2024, date last accessed). (in Japanese).

[3] Jointly Written by Nikkei Inc. One Year after the Return of Residents to Futaba-Machi, Fukushima, 86 People Are Living in the Town, One Step at a Time toward Recovery. Nihon Keizai Shimbun, 1-3-7 Otemachi, Chiyoda-ku, Tokyo. https://www.nikkei.com/article/DGXZQOUE30DFF0Q3A830C2000000/ (29 January 2024, date last accessed). (in Japanese).

[4] Matsunaga H , Orita M, Oishi K, et al. Intention to return in residents of Okuma and its characteristics: the evacuation order was lifted eight years after the Fukushima Daiichi Nuclear Power Station accident. J Radiat Res 2021;62:868–70. 10.1093/jrr/rrab058. 34313302 PMC8438246

[5] Jozuka E , Yeung J. Fukushima town lifts evacuation order, allowing former residents to return 11 years after nuclear disaster. CNN World; 2:01 AM EDT, Tue August 30, 2022. https://edition.cnn.com/2022/08/30/asia/futaba-fukushima-nuclear-evacuation-order-intl-hnk/index.html (29 January 2024, date last accessed).

[6] ICRP . Application of the Commission’s Recommendations to the Protection of People Living in Long-Term Contaminated Areas after a Nuclear Accident or a Radiation Emergency. ICRP Publication 111., Vol. 39. 350 Albert StreetSuite 410 Ottawa, OntarioK1R 1A4 CANADA, Ann ICRP, 2009. 10.1016/j.icrp.2009.09.008 20472181

[7] Xiao X , Matsunaga H, Orita M, et al. Assessment of radiation risk perception and interest in tritiated water among returnees to and evacuees from Tomioka town within 20 km of the Fukushima Daiichi nuclear power plant. Int J Environ Res Public Health 2023;20:2690. 10.3390/ijerph20032690. 36768061 PMC9915426

[8] Health and Welfare Division, Futaba Town Office. Health & Medical Notices: “Futaba-Machi Clinic Opens!”, 73-4 Chonishi, Nagatsuka, Futaba Town, Futaba District, Fukushima Prefecture. https://www.town.fukushima-futaba.lg.jp/10354.htm (20 March 2024, date last accessed). (in Japanese).

[9] Family Registration and Taxation Division, Futaba Town Office. Population and Number of Households in Futaba-Machi (since the Great East Japan Earthquake). 73-4 Chonishi, Nagatsuka, Futaba Town, Futaba District, Fukushima Prefecture. https://www.town.fukushima-futaba.lg.jp/5873.htm (20 March 2024, date last accessed). (in Japanese).

[10] Family Registration and Taxation Division, Futaba Town Office. Evacuation Status (as of May 31, 2022). 73-4 Chonishi, Nagatsuka, Futaba Town, Futaba District, Fukushima Prefecture. https://www.town.fukushima-futaba.lg.jp/10062.htm (20 March 2024, date last accessed). (in Japanese).

[11] Scientific Committee on the Effects of Atomic Radiation (UNSCEAR) . Sources, Effects and Risks of Ionizing Radiation. 2021. Vienna International Centre Wagramer Straße 5 P.O. Box 500. Building E A-1400 Vienna, AUSTRIA. https://www.unscear.org/unscear/uploads/documents/unscear-reports/UNSCEAR_2020_21_Report_Vol.II.pdf (29 January 2024, date last accessed).

[12] Hande V , Orita M, Matsunaga H, et al. Changes in the intention to return and the related risk perception among residents and evacuees of Tomioka town 11 years after the Fukushima nuclear accident. Disaster Med Public Health Prep 2023;17:e386,1–8. 10.1017/dmp.2023.58. 37165606

[13] Liu M , Matsunaga H, Orita M, et al. Risk perception of genetic effects and mental health among residents of Kawauchi village, 10 years after the Fukushima Daiichi nuclear power plant accident. J Radiat Res 2022;63:261–3. 10.1093/jrr/rrab108. 34999893 PMC8944293

[14] Sugimoto A , Nomura S, Tsubokura M, et al. The relationship between media consumption and health-related anxieties after the Fukushima Daiichi nuclear disaster. PLoS ONE 8(8):e65331. 10.1371/journal.pone.0065331. PMC3743804 23967046

[15] Hande V , Orita M, Matsunaga H, et al. Comparison of quality of life between elderly and non-elderly adult residents in Okuma town, Japan, in a post-disaster setting. PLoS ONE 18(2):e0281678. 10.1371/journal.pone.0281678. PMC9928109 36787311

[16] Kelley K , Clark B, Brown V, et al. Good practice in the conduct and reporting of survey research. Int J Qual Health Care 2003;15:261–6. 10.1093/intqhc/mzg031. 12803354

[17] Horikoshi N , Iwasa H, Yasumura S, Maeda M. The characteristics of non-respondents and respondents of a mental health survey among evacuees in a disaster: the Fukushima health management survey. Fukushima J Med Sci 2017;63:152–9. 10.5387/fms.2017-03. 29237989 PMC5792499

