# Supplementary material for: Risk perception in long-term evacuees of Futaba town, Fukushima: a cross-sectional study reveals greater concerns outside the prefecture, 12 years after the accident
Source: J Radiat Res. 2024 Jun 11;65(4):549–54. doi: 10.1093/jrr/rrae039 (PMC11262855; doi:10.1093/jrr/rrae039)
Supplement: TableS1-clean_rrae039 [file tables1-clean_rrae039.docx]

**Table S1.** Sociodemographic characteristics, risk perception, and expectations for town revival of study participants according to anxiety about genetic effects.

|  |  | **Anxiety about genetic effects.** | | p value | |
| --- | --- | --- | --- | --- | --- |
|  |  | Yes (n =242) | No (n = 162) | |  |
| Sex | Male | 118(48.8%) | 93(57.4%) | | 0.088 |
|  | Female | 124 (51.2%) | 69 (42.6%) | |  |
| Age (y) | < 60 | 59 (24.4%) | 28 (17.3%) | | 0.089 |
|  | ≥ 60 | 183(75.6%) | 134 (82.7%) | |  |
| Employed | Yes | 62 (25.6%) | 40 (24.7%) | | 0.883 |
|  | No | 180(74.4%) | 122 (75.3%) | |  |
| Evacuation location | Outside Fukushima | 93(38.4%) | 37(22.8%) | | 0.001* |
|  | Inside  Fukushima | 149(61.6%) | 125(77.2%) | |  |
| Regular hospital visits | Yes | 196 (81.0%) | 134(82.7%) | | 0.661 |
|  | No | 46 (19.0%) | 28 (17.3%) | |  |
| Living with children aged < 18 y | Yes | 42 (17.4%) | 16 (9.9%) | | 0.036* |
|  | No | 200 (82.6%) | 146(90.1%) | |  |
| Intention to return | Intended | 18 (7.4%) | 24 (14.8%) | | 0.007* |
|  | Unsure | 86 (35.5%) | 38 (23.5%) | |  |
|  | Not intended | 138 (57.0%) | 100 (61.7%) | |  |
| Mental Component Summary | < 50 | 165 (68.2%) | 71 (43.8%) | | ＜0.001* |
|  | ≥ 50 | 77 (31.8%) | 91 (56.2%) | |  |
| **Risk perception variable** |  |  |  | |  |
| Aware of radiation consultation center in Futaba town | Yes  No | 72 (29.8%)  170 (70.2%) | 97 (59.9%)  65 (40.1%) | | ＜0.001* |
| Wish to acquire knowledge about nuclear radiation | Yes  No | 166 (68.6%)  76 (31.4%) | 92 (56.8%)  70 (43.2%) | | 0.015* |
| Anxious about discharge of treated water | Yes  No | 199 (82.2%)  43 (17.8%) | 64 (39.5%)  98 (60.5%) | | ＜0.001* |
| Wish to acquire knowledge about treated water | Yes  No | 192 (79.3%)  50 (20.7%) | 89 (54.9%)  73 (45.1%) | | ＜0.001* |
| Anxious about drinking tap water in Futaba town | Yes  No | 214 (88.4%)  28 (11.6%) | 67 (41.4%)  95 (58.6%) | | ＜0.001* |
| Anxious about the health effects of radiation exposure | Yes  No | 200 (82.6%)  42 (17.4%) | 27 (16.7%)  135 (83.3%) | | ＜0.001* |
| **Expectations for town revival** |  |  |  | |  |
| Expect redevelopment of workplaces | Yes  No | 117 (48.3%)  125 (51.7%) | 114 (70.4%)  48 (29.6%) | | ＜0.001* |
| Expect redevelopment of farmland | Yes  No | 94 (38.8%)  148 (61.2%) | 107 (66.0%)  55 (34.0%) | | ＜0.001* |
| Expect rebuilding in residential areas | Yes  No | 112 (46.3%)  130 (53.7%) | 113 (69.8%)  49 (30.2%) | | ＜0.001* |
| Expect town revival | Yes  No | 162 (66.9%)  80 (33.1%) | 135 (83.3%)  27 (16.7%) | | ＜0.001* |

Note: chi-square test. *: P < 0.05; **: P < 0.001.
