# Supplementary material for: Risk perception in long-term evacuees of Futaba town, Fukushima: a cross-sectional study reveals greater concerns outside the prefecture, 12 years after the accident
Source: J Radiat Res. 2024 Jun 11;65(4):549–54. doi: 10.1093/jrr/rrae039 (PMC11262855; doi:10.1093/jrr/rrae039)
Supplement: TableS2-clean_rrae039 [file tables2-clean_rrae039.docx]

**Table S2.** Sociodemographic characteristics, risk perception, and expectations for town revival of study participants according to anxiety about health effects.

|  |  | **Anxiety about health effects** | | p value | |
| --- | --- | --- | --- | --- | --- |
|  |  | Yes (n =227) | No (n = 177) | |  |
| Sex | Male | 113(49.8%) | 98(55.4%) | | 0.265 |
|  | Female | 114 (50.2%) | 79 (44.6%) | |  |
| Age (y) | < 60 | 52 (22.9%) | 35 (19.8%) | | 0.447 |
|  | ≥ 60 | 175(77.1%) | 142 (80.2%) | |  |
| Employed | Yes | 53 (23.3%) | 49 (27.7%) | | 0.320 |
|  | No | 174(76.7%) | 128 (72.3%) | |  |
| Evacuation location | Outside Fukushima | 86(37.9%) | 44(24.9%) | | 0.005* |
|  | Inside  Fukushima | 141(62.1%) | 133(75.1%) | |  |
| Regular hospital visits | Yes | 188 (82.8%) | 142(80.2%) | | 0.504 |
|  | No | 39 (17.2%) | 35 (19.8%) | |  |
| Living with children aged < 18 y | Yes | 38 (16.7%) | 20 (11.3%) | | 0.122 |
|  | No | 189 (83.3%) | 157(88.7%) | |  |
| Intention to return | Intended | 16 (7.0%) | 26 (14.7%) | | 0.010* |
|  | Unsure | 80 (35.2%) | 44 (24.9%) | |  |
|  | Not intended | 131 (57.7%) | 107 (60.5%) | |  |
| Mental Component Summary | < 50 | 156 (68.7%) | 80 (45.2%) | | ＜0.001* |
|  | ≥ 50 | 71 (31.3%) | 97 (54.8%) | |  |
| **Risk perception variable** |  |  |  | |  |
| Aware of radiation consultation center in Futaba town | Yes  No | 76 (33.5%)  151 (66.5%) | 93 (52.5%)  84 (47.5%) | | ＜0.001* |
| Wish to acquire knowledge about nuclear radiation | Yes  No | 163 (71.8%)  64 (28.2%) | 95 (53.7%)  82 (46.3%) | | ＜0.001* |
| Anxious about discharge of treated water | Yes  No | 194 (85.5%)  33 (14.5%) | 69 (39.0%)  108 (61.0%) | | ＜0.001* |
| Wish to acquire knowledge about treated water | Yes  No | 183 (80.6%)  44 (19.4%) | 98 (55.4%)  79 (44.6%) | | ＜0.001* |
| Anxious about drinking tap water in Futaba town | Yes  No | 206 (90.7%)  21 (9.3%) | 75 (42.4%)  102 (57.6%) | | ＜0.001* |
| Anxious about the genetic effects of radiation exposure | Yes  No | 200 (88.1%)  27 (11.9%) | 42 (23.7%)  135 (76.3%) | | ＜0.001* |
| **Expectations for town revival** |  |  |  | |  |
| Expect redevelopment of workplaces | Yes  No | 114 (50.2%)  113 (49.8%) | 117 (66.1%)  60 (33.9%) | | ＜0.001* |
| Expect redevelopment of farmland | Yes  No | 90 (39.6%)  137 (60.4%) | 111 (62.7%)  66 (37.3%) | | ＜0.001* |
| Expect rebuilding in residential areas | Yes  No | 112 (49.3%)  115 (50.7%) | 113 (63.8%)  64 (36.2%) | | 0.004* |
| Expect town revival | Yes  No | 157 (69.2%)  70 (30.8%) | 140 (79.1%)  37 (20.9%) | | 0.025* |

Note: chi-square test. *: P < 0.05; **: P < 0.001.
